# Serum myosin-binding protein c levels: a new marker for exclusion of preterm birth?

**DOI:** 10.55730/1300-0144.5717

**Published:** 2023-09-09

**Authors:** Elif YILDIZ, Fatma Ketenci GENCER, Burcu TİMUR, Hakan TİMUR

**Affiliations:** 1 Department of Obstetrics and Gynecology, Gaziosmanpaşa Training and Research Hospital, University of Health Sciences, İstanbul, Turkiye; 2 Department of Obstetrics and Gynecology, Ordu University Training and Research Hospital, Ordu, Turkiye

**Keywords:** Myosin-binding protein C, preterm delivery, imminent labor prediction, uterine myoelectrical activity, uterine contraction

## Abstract

**Background/aim:**

To evaluate whether there is a relationship between serum myosin-binding protein C (MyBP-C) levels measured in the first trimester and the timing of delivery, and, if a relationship is detected, the potential of this relationship in distinguishing between preterm and term labor.

**Materials and methods:**

This prospective case-control study was conducted with 701 pregnant women who applied to the Obstetrics Outpatient Clinic of Gaziosmanpaşa Training and Research Hospital in the first trimester, between 11 and 14 gestational weeks. MyBP-C serum samples from the first trimester were stored under appropriate conditions until the time of delivery. Of these pregnant women, 628 completed the study. According to the delivery time, the pregnant women were divided into two groups, as those who delivered prematurely before 37 weeks and those who gave birth at term. The case group comprised 45 women who gave birth prematurely, while 583 women gave birth at term. A control group was formed with 45 pregnant women of the same age, who were selected by randomization using a simple random sampling method from the 583 pregnant women. The MyBP-C levels were measured and compared from the first-trimester serum materials of both groups.

**Results:**

The MyBP-C levels of the preterm delivery group were significantly higher than those of the term delivery control group (4.51 ± 1.69 vs. 3.09 ± 1.44 pg/mL, respectively; p < 0.001). Receiver operating characteristic (ROC) curve analysis showed that the MyBP-C levels in the first trimester with a cut-off value of 4.76 ng/dL indicated women with preterm delivery with a sensitivity of 42.22% and specificity of 95.56% (AUC: 0.734, 95% CI: 0.630–0.822). The overall differential diagnosis performance of the MyBP-C level for preterm delivery was determined as 73.4% (p < 0.001). The MyBP-C levels were found to be significantly higher both in the early preterm group compared with the late preterm group (p < 0.001), and in those with premature rupture of membranes (PROM) compared with those without (p < 0.001).

**Conclusion:**

The preterm delivery group exhibited high serum MyBP-C levels in the serum samples taken in the first trimester. First-trimester serum MyBP-C levels seem to be a simple and easy way to exclude preterm delivery risk in a significant manner. In addition, levels are significantly higher for early preterm compared with late preterm and early PROM compared with intact membranes.

## 1.Introduction

Preterm labor is defined as regular contractions between 20 and 37 weeks of gestation that cause dilation of the cervix [1]. It is one of the leading causes of neonatal morbidity and death worldwide [2]. Moreover, preterm labor introduces additional serious problems for the newborn itself, the family, caregivers, and the national health system [3,4]. Neonatal intensive care admissions are among the most expensive types of hospitalizations [5]. All of these negative effects increase at a much more serious rate as the birth week gets earlier. For these reasons, it is very important to diagnose premature birth correctly and to predict it, if possible.

Myosin-binding protein C (MyBP-C) makes up about 2% of myofibrillar proteins and is a member of the thick myosin filaments family [6]. Currently, three isoforms have been identified, which are cardiac, slow skeletal, and fast skeletal, and mutations in these isoforms have been associated with some diseases such as tibial muscular dystrophy and familial hypertrophic cardiomyopathy [7]. Its working principle is to provide the organization and regulation of thick filaments and to ensure the formation of cross-bridges by providing a direct connection between actin and myosin [8,9].

Although it is generally accepted that MyBP-C can interact with the three filament systems (thick, thin, and titin filaments) within the sarcomere, the exact nature of these interactions and the functional implication of the modified binding remains unclear [10]. The presence of MyBP-C in striated and cardiac muscle has been shown, but there is no clear association with the contraction of smooth muscle. However, although it is known that smooth muscle contraction is stronger than in striated muscle, the mechanism that causes this has not yet been fully elucidated [11]. Harris and Warshaw presented evidence from an in vitro motility analysis that functional cycles were not different for smooth and skeletal muscle myosin [12]. Based on these and similar common features of MyBP-C, which is accepted as an important modulator of contraction [13], it is our belief that an undetected MyBP-C isoform may also be effective on smooth muscle contraction and could even be effective in the formation of stronger cross-links.

Accordingly, it was aimed to investigate the relationship between first-trimester serum MyBP-C levels and preterm labor, which is usually caused by contractions of the uterus, which contains smooth muscle in its structure.

## 2. Materials and methods

This prospective case-control study was conducted at Health Sciences University, Gaziosmanpaşa Training and Research Hospital, between May 2022 and March 2023. The study was approved by the institutional review board and ethics committee, complying with the Declaration of Helsinki.

Before starting the study, a power analysis was performed. For the comparison of two independent groups (patient and control), it was found appropriate to include 42 people in each group and a total of 84 people with 80% power, 5% type I error, and at least 0.6 effect size. The calculation was made using the G*Power 3.1.9.4 program. The incidence of preterm birth can vary between 7.7% and 14.8% [14]. In the calculations based on this, it was predicted that a minimum of 580 patients would be included in the study, and it was aimed as the end point of the study to reach 42 in the case group. A total of 701 singleton pregnant women who applied for a first trimester screening test, at weeks 11–14 of gestation, were included in the study. The inclusion criteria were being 18 years of age or older, being in the first trimester of pregnancy, at weeks 11–14 of gestation, and having a fetal heartbeat as confirmed through ultrasound. Gestational week was calculated by combining the last menstrual period and ultrasonographic data. Pregnant women who had no fetal heartbeat, had placenta previa, fetal anomalies, and had a pregnancy greater than the first trimester (>14 weeks) at the time of first admission were excluded from the study. The demographic characteristics of all of the participants who agreed to participate in the study were recorded. Body mass index (BMI) was calculated by dividing the weight by the square of the height (kg/m^2^). The World Health Organization (WHO) describes obesity BMI scores as higher than 30 kg/m^2^ [15]. Obstetric anamnesis and ultrasonographic examinations were performed. All ultrasound examinations were performed using a 4.5–16.5-MHz transabdominal probe or with a 5–9-MHz transvaginal transducer (Mindray DC8 Expert, Wauwatosa). In cases where it was difficult to visualize the fetus (such as with a high BMI), the examination was performed vaginally (5–9 MHz). On the same day, the patients were asked to give blood samples for the study. Blood samples were collected from the antecubital veins while the patients were in a sitting position. After centrifugation at 4000 rpm for 10 min at 4 °C, the supernatant serum samples were transferred to 1.5-mL Eppendorf tubes. The serum was stored at –80 °C. All of the samples were thawed only once before use. Variables obtained from the serum analysis were thyroid stimulating hormone (TSH), aspartate aminotransferase (AST), alanine aminotransferase (ALT), urea, creatinine, and MyBP-C.

Measurements of the TSH variables were obtained using a Uni Cel DxI 800 chemistry system (Roche HITACHI Cobas c 501; Roche Diagnostics, Basel, Basel-Stadt, Switzerland). Hemogram levels were measured using a Mikromed Mindray BC-6800. Urea, creatinine, AST, and ALT measurements were obtained using a Uni Cel DxI 800 chemistry system (Roche HITACHI Cobas). MyBP-C levels were measured in a microplate reader RT 2100 C and microplate washer RT 2600 C devices with a commercially available kit (Bostonchem, BLS-3648Hu MyBP-C, ELISA kit).

The participants were followed-up throughout their pregnancies until the time of delivery. Patients who gave birth in other centers were also contacted via telephone and the national health information registration system (e-nabız). The patients were grouped according to their weeks of birth. Preterm delivery was defined as delivery before 37 completed weeks of gestation (<259 days). Women who gave birth after 20 weeks and before 37 weeks were included in the case group, and women who gave birth at 37 weeks and later were included in the control group.

The study was started with 701 women, considering losses, those who could not complete the study, and patients whose information could not be accessed for any reason, 628 women completed the study. Of these women, 45 gave birth prematurely, and 583 women gave birth at term. Of the women who gave birth prematurely, 45 constituted the study group; 583 women with term delivery were randomized using a simple random sampling method and a control group was formed by determining 45 participants. The data of both groups were compared and analyzed.

### 2.1. Statistical analysis

All statical analyses were performed using IBM SPSS Statistics for Windows 21.0 (IBM Corp., Armonk, NY, USA) and MedCalc version 20.104 (MedCalc Software Ltd., Ostend, Belgium). Normality of the continuous variables was evaluated using the Shapiro–Wilk test. Quantitative variables were expressed as the mean ± standard deviation and qualitative variables were expressed as percentages. The Mann–Whitney U and Student’s t-tests were used to compare two independent groups. The Student’s t-test was used to compare variables with normal distribution, and quantitative variables with nonnormal distribution were compared using the Mann–Whitney U test. The chi-squared test was used in the analysis of categorical data. p ≤ 0.05 was considered statistically significant. Receiver operating characteristic (ROC) curve analyses were used to determine the predictive role of MyBP-C for preterm delivery. The sensitivity, specificity, positive predictive values (PPV), and negative predictive values (NPV) of the MyBP-C were calculated for the estimation of the development of preterm delivery.

### 2.2. Ethical statement

The study protocol was approved by the Ethics Committee of Health Sciences University, Gaziosmanpaşa Training and Research Hospital (no.: 46, dated: 15/03/2022), and was conducted according to the principles of the Helsinki Declaration. Written informed consent was obtained from all of the patients. The clinical trial registration number of the study is NCT05513768.

## 3. Results

A total of 701 pregnant women who presented to the Pregnancy Outpatient Clinic of Gaziosmanpaşa Training and Research Hospital for routine pregnancy follow-ups were included in the study. Women whose first-trimester blood samples were taken were followed-up until the time of delivery. Thirty-eight pregnant women were excluded from the study due to delays in their pregnancy follow-ups and inaccessibility to birth information. Moreover, 14 participants withdrew from the study at their own request. The pregnancies of 21 women were excluded because they ended in abortion before 20 weeks.

Clinical and demographic parameters of 45 patients with preterm birth and 45 age-matched term birth controls are shown in Table 1. There was no statistical difference between the groups regarding age, gravidity, parity, number of abortions, BMI, smoking, cesarean and normal birth rates, baby sex, urea, creatinine, AST, ALT, TSH, and hemoglobin levels. However, a statistically significant difference was found in terms of the birth week, fetal birth weight, and MyBP-C levels between the groups.

Of the women, 4 (8.9%) in the study group received 2 doses of steroid prophylaxis in the antenatal period, 2 (4.4%) received a single dose of steroid prophylaxis, and 39 (86.7%) received no antenatal steroid prophylaxis. In the study group, 8 (17.8%) women received antibiotic treatment in the antenatal period, and 37 (82.2%) received no antibiotic treatment. Premature rupture of membranes (PROM) was observed in 14 (31.1%) of the women in the preterm delivery group. PROM was not observed in 31 (68.9%) women.

**Figure 1:**
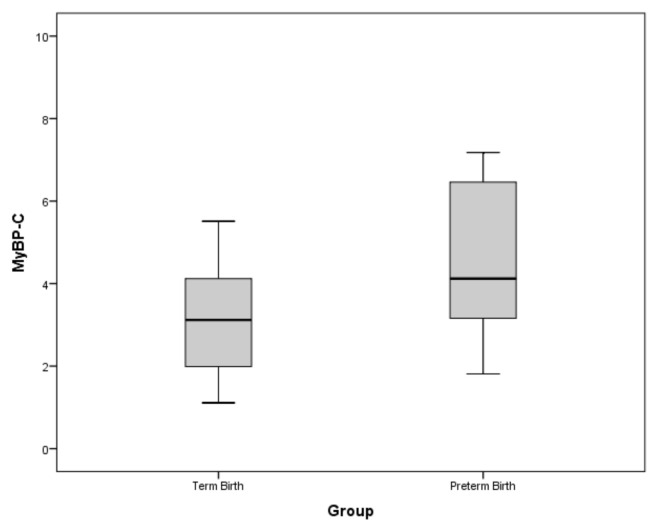
Comparison of the plasma MyBP-C levels among the study subjects.

As shown in Figure 1 and Table 2, the MyBP-C levels of the preterm birth group were significantly higher than in the control group (p < 0.001).

**Figure 2:**
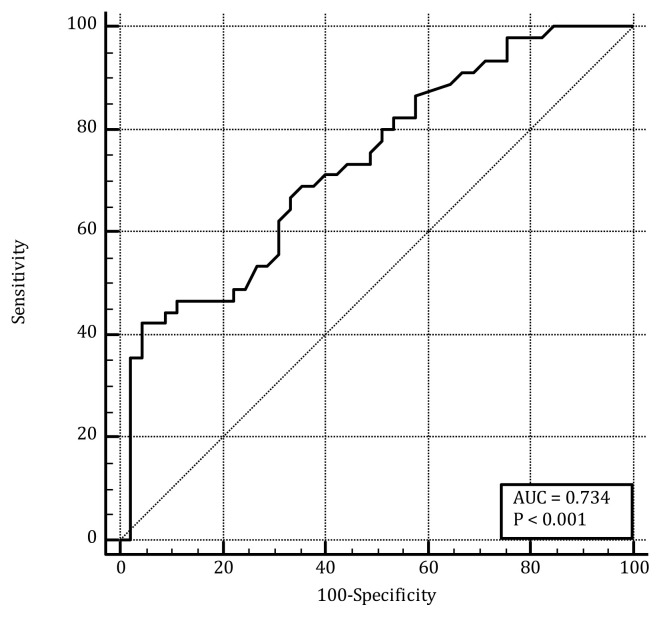
ROC for the predictive value of the MyBP-C to predict preterm birth values.

The cut-off value for separating preterm and normal birth for the outcome variable was 4.76. Using the cut-off value, the MyBP-C level measured in the first-trimester serum could distinguish 42.22% of preterm babies and 95.56% of normal deliveries. The general discrimination power of MyBP-C levels measured in the first-trimester serum for preterm birth was 73.4%, which was statistically significant (p < 0.001) (Figure 2, Table 3).

Premature birth is classified as late or preterm birth according to the week of onset. Late preterm birth is classified as 34–37 weeks. Premature birth is defined as the birth of the baby before week 34 (20 – 33 + 6) [16]. In the current study, the difference was statistically significant when the preterm delivery group was divided into two subgroups, as early preterm delivery and late preterm delivery, and when MyBP-C levels were examined. In addition, the MyBP-C levels were significantly higher in the women with PROM than in those with preterm birth without PROM (Table 2).

## 4. Discussion

This study is the first in the literature to investigate the relationship between MyBP-C levels in serum in the first trimester and the timing of delivery. The main results of the present study indicate that the mean serum MyBP-C level is significantly higher in patients with preterm delivery compared with healthy pregnant women who give birth at term (p < 0.001). In addition. MyBP-C levels are significantly higher for early preterm compared with late preterm and early PROM compared with intact membranes.

There are many studies on the mechanisms that initiate labor and the conditions that cause preterm birth, but the information on this subject remains unclear. Most studies on predicting the timing of birth aim to predict preterm birth because the more critical issue is about being able to distinguish term-preterm regarding the baby, rather than the day of the birth. Because if we can predict it in the early trimester, progesterone treatment can be started in the early period. Various markers have been screened to predict preterm birth, tests have been tried to be developed, and different mechanisms have been proposed. When considering the literature, the opinion that preterm birth occurs as a result of inflammation is dominant [17]. Considering that the bronchi with smooth muscle in their structure contract as a result of inflammation, as in asthma, it is not improbable for the contractions in the uterus to start due to inflammation [18]. In the literature, the increase in MyBP-C levels in heart attacks that develop due to chronic inflammation creates the idea that MyBP-C levels may also be effective in the contraction of the uterus through the same mechanism [19]. In the current study, the fact that the MyBP-C levels were significantly higher in the first trimester in patients diagnosed as having preterm birth compared with those who gave birth at term, and that the MyBP-C levels were significantly higher in the women who did not have PROM, may indicate the relationship between preterm labor and preterm labor triggered by an early-onset chronic inflammation. In addition, the MyBP-C levels of 8 women who gave birth before 34 weeks were significantly higher than those who delivered late preterm, between 34 and 37 weeks, which leads to the conclusion that it is directly related to the severity of preterm labor. However, this result needs to be supported by new studies including more patients because the number of premature preterm births was low in the current study. As the gestational week gets earlier, the negative effects on the newborn increase exponentially. Accordingly, predicting preterm labor is very valuable, but predicting early preterm birth is more valuable.

If this idea is supported by more participatory studies, it is our hope that great advantages will be provided in preterm labor in terms of actions under 34 weeks, which are even riskier, and this will help mitigate the negative effects for the newborn itself, and of the familial and national burden. Given that the prepregnancy values were not known in the current study, it is not possible to know whether the MyBP-C levels that increased in the preterm birth group were higher before, or if they started to increase after conception. In addition, in the present study, the first-trimester values were only measured once. The values in other trimesters were not evaluated and it was not possible to show if the changes in repeated measurements would progress from week to week until delivery. This may be the subject of future studies. Again in the current study, the MyBP-C levels were found to be significantly higher when the group with PROM was compared with the group without. It is known that PROM brings many negative factors such as chorioamnionitis, sepsis, fetal infections, increased cesarean section rates, long-term use of antibiotics, prolonged hospital stay, and additional cost [20,21]. For this reason, foreseeing and taking precautions will be of great benefit to the mother as well as the newborn. The fact that the MyBP-C levels rises in PROM is very valuable in that it gives rise to the hope that it can be used as a predictive novel marker in PROM and deserves to be evaluated with further studies. Predicting preterm labor, informing pregnant women of potential risk in this regard, and keeping them under closer follow-up will be promising for these patients. At this point, if a strong correlation between preterm labor and MyBP-C levels can be proven, it will be useful for both patients and physicians to have a simple determination of MyBP-C levels in diagnosis and treatment. The data herein were cross-sectional and too weak to suggest a predictive role of MyBP-C in terms of delivery and neonatal health in patients with preterm labor, but the results need to be considered in this regard.

There were some limitations to the study. First, it was a single-center study in which only a limited number of pregnant women who presented to the outpatient clinic were included. Another limitation was that when choosing the control group, simple randomization was chosen and a certain number of participants were included in the control group, not the entire group. This may not have reflected the characteristics of the whole group. When the difference in the MyBP-C levels between the case and control groups is considered, it showed a high specificity but had a low sensitivity of 42.22%. This indicates the higher discriminating power in term birth pregnancies. In addition, no data regarding the health problems of the newborn babies were collected in the study.

It is unknown whether there is a link between MyBP-C levels and neonatal prognosis. This may be the subject of new studies. The current study can be a source for calculating sample sizes for future studies to make more precise associations because there has never been a study investigating the relationship between preterm birth and MyBP-C levels. In vivo studies, in particular, are needed to clarify the physiopathological relationship between MyBP-C and uterine smooth muscle contraction and to elucidate the mechanism.

As a result of this study, it was found that the MyBP-C levels of the preterm delivery group were significantly higher than in the term delivery group. The levels were higher in the early preterm group than in the late preterm group. Significantly higher levels were found in the group with PROM compared with the group without. All of these results raise the possibility that MyBP-C may be a novel marker for excluding preterm labor in terms of the timing of delivery, predicting the severity of preterm labor, and whether PROM develops while evaluating a pregnancy in the first trimester. This study is the first on this subject. The relationship of MyBP-C with the mechanisms that cause labor should be investigated through further studies with a greater sample size.

## Figures and Tables

**Table 1:** Clinical characteristics of the study participants in each group.

	Preterm birth			Term birth			
	Mean ± SD	Median [IQR]	Min–max	Mean ± SD	Median [IQR]	Min–max	p-value
Birth week	35.8 ± 1.09	36.14 [35.86–36.43]	32–36.71	39.17 ± 0.8	39.29 [38.71–40]	37.71–40.43	**<0.001**
Baby weight (g)	2629.84 ± 365.44	2700 [2400–2900]	1658–3100	3279.64 ± 217.06	3245 [3100–3450]	2990–3700	**<0.001**
Age (years)	28.33 ± 4.37	29 [26–31.5]	17–36	27.91 ± 4.55	28 [24–31]	20–40	0.655*
Gravida (n)	3.07 ± 1.67	3 [2–4]	1–8	2.69 ± 1.49	3 [2–3]	1–8	0.254
Parity (n)	1.71 ± 1.38	2 [0–2.5]	0–5	1.47 ± 1.31	1 [0.5–2]	0–5	0.298
Abortus (n)	0.31 ± 0.63	0 [0–0]	0–2	0.22 ± 0.6	0 [0–0]	0–3	0.411
Curettage (n)	0.04 ± 0.3	0 [0–0]	0–2	0.02 ± 0.15	0 [0–0]	0–1	0.987
Height (cm)	159.13 ± 5.25	160 [156–163]	150–170	161.09 ± 5.8	160 [158–165]	150–172	0.182
Weight (kg)	73.67 ± 12.86	75 [62.5–83]	53–100	72.56 ± 15.78	70 [61.5–81.5]	50–118	0.458
BMI (kg/m^2^)	29.1 ± 4.97	29.69 [25.06–32.37]	19.95–41.33	27.91 ± 5.6	26.22 [24.17–31.62]	17.72–42.31	0.173
Urea (mg/dL)	15.36 ± 5.45	14 [12–18]	9–30	15.2 ± 4.41	14 [12–17]	8–30	0.739
Creatinine (mg/dL)	0.7 ± 1.27	0.48 [0.43–0.58]	0.37–9	1.23 ± 2.28	0.44 [0.42–0.54]	0.33–9	0.106
AST (IU/L)	15.87 ± 3.24	15 [14–18]	11–23	15.24 ± 4.25	14 [12–17]	9–34	0.197
ALT (IU/L)	11.09 ± 4.15	10 [8–13]	6–24	10.02 ± 3.95	9 [7.5–13]	3–22	0.194
TSH	2.28 ± 2.2	1.68 [1.31–2.85]	0.56–15	1.93 ± 1.15	1.7 [1.31–2.2]	0.12–6	0.743
Hgb (g/dL)	11.18 ± 1.05	11.3 [10.4–11.8]	8.9–13.9	11.16 ± 1.04	11 [10.5–11.9]	8.9–13.5	0.928*

p-value: Mann–Whitney U test,

* Student’s t-test.

**Table 2:** Receiver operating curve (ROC) for the predictive value of MyBP-C to predict preterm birth values.

Criterion	AUC (95% CI)	Sensitivity (95% CI)	Specificity (95% CI)	p-value
>4.76	0.734 (0.630–0.822)	42.22 (27.7–57.8)	95.56 (84.9–99.5)	<0.001

**Table 3:** MyBP-C levels.

	Preterm birth			Term birth			
	Mean ± SD	Median [IQR]	Min–max	Mean ± SD	Median [IQR]	Min–max	p-value
MyBP-C	4.51 ± 1.69	4.12 [3.14–6.47]	1.81–7.18	3.09 ± 1.44	3.12 [1.94–4.14]	1.11–8.8	**<0.001**
	Early preterm (n = 8) (20 – 33 + 6 weeks)			Late preterm (n = 37) (34 – 36 + 6 weeks)			
	Mean ± SD	Median [IQR]	Min–max	Mean ± SD	Median [IQR]	Min–max	p-value
MyBP-C	3.42 ± 1.42	3.21 [2.26–3.95]	1.93–6.39	4.74 ± 1.67	4.56 [3.36–6.49]	1.81–7.18	**<0.001**
	PROM (+) n = 31			PROM (−) n = 14			
	Mean ± SD	Median [IQR]	Min–max	Mean ± SD	Median [IQR]	Min–max	p-value
MyBP-C	3.64 ± 1.16	3.56 [2.56–4.36]	1.81–5.8	6.41 ± 0.97	6.65 [6.47–6.81]	3.11–7.18	**<0.001**

p-value: Mann–Whitney U test.
